# Mobility impairment and life satisfaction in the Northern Region of Malawi

**DOI:** 10.4102/ajod.v11i0.1013

**Published:** 2022-09-22

**Authors:** Jared M. Alswang, William B. Belshe, Dexter Killi, Weston Bandawe, Erin S. Silliman, Aaron C. Bastian, Brooke K. Upchurch, Megan F. Bastian, Sierra M. Pinal, Mark B. Klein, Bertha Ndhlozi, Mauricio Silva, John Chipolombwe, Rachel M. Thompson

**Affiliations:** 1 Harvard Medical School, Harvard University, Boston, MA,United States of America; 2 David Geffen School of Medicine, University of California Los Angeles, Los Angeles, CA, United States of America; 3 Department of Physiotherapy, Mzuzu Central Hospital, Mzuzu, Malawi; 4 Department of Physiotherapy, St. John’s Hospital, Mzuzu, Malawi; 5 School of Medicine, Boston University, Boston, MA, United States of America; 6 College of Osteopathic Medicine, New York Institute of Technology, Glen Head, NY, United States of America; 7 Dell Medical School, University of Texas at Austin, Austin, TX, United States of America; 8 School of Medicine, Saint Louis University, Saint Louis, MO, United States of America; 9 Department of Orthopedic Surgery, David Geffen School of Medicine, University of California Los Angeles, Los Angeles, CA, United States of America; 10 Center for Cerebral Palsy, Orthopaedic Institute for Children, Los Angeles, CA, United States of America; 11 Dornsife College of Letters, Arts and Science, University of Southern California, Los Angeles, CA, United States of America; 12 Malawi Against Physical Disabilities, Rumphi, Malawi; 13 Orthopaedic Institute for Children, Los Angeles, CA, United States of America; 14 Department of Internal Medicine, Mzuzu Central Hospital, Mzuzu, Malawi

**Keywords:** mobility impairment, life satisfaction, physical activity, mobility assistive devices, physical disability

## Abstract

**Background:**

There exist many psychosocial sequelae associated with mobility impairment, especially in low-resource settings where access to mobility assistive devices is limited.

**Objectives:**

This study aims to (1) define the burden and presenting aetiologies of mobility impairment in the rural Northern Region of Malawi and (2) assess the relationship between physical disability, life satisfaction and access to mobility aids.

**Methods:**

At mobility device donation clinics throughout the Northern Region of Malawi, adults living with mobility impairment were surveyed with a demographic questionnaire and a series of validated surveys to assess their physical activity levels (Global Physical Activity Questionnaire [GPAQ]), degree of mobility impairment (Washington Group Extended Set Questions on Disability) and life satisfaction (patient-reported outcomes measurement information systems satisfaction with participation in social roles and general life satisfaction).

**Results:**

There were 251 participants who qualified for inclusion, of which 193 completed all surveys. Higher physical activity scores were positively correlated with increased life satisfaction: (1) satisfaction with participation in social roles (0.481, *p* < 0.0001) and (2) general life satisfaction (0.230, *p* < 0.001). Respondents who had previously used a formal mobility device reported 235.5% higher physical activity levels ([139.0%, 333.0%], *p* = 0.006), significantly higher satisfaction with participation in social roles ([0.21, 6.67], *p* = 0.037) and equivocally higher general life satisfaction ([−1.77, 3.84], *p* = 0.470).

**Conclusion:**

Disability and mental health do not exist in isolation from one another. Given the positive correlations between formal mobility device usage and both physical activity and life satisfaction, interventions that increase access to mobility-assistive devices in undertreated populations are imperative.

**Contribution:**

This study contributes to the understanding of the complex relationship between physical disability, access to mobility aids, and life satisfaction. Results from this study suggest the potential benefit that increasing access to mobility aids may have in improving the quality of life of mobility impaired persons in resource-limited settings, such as the Northern Region of Malawi.

## Introduction

The global burden of disability is increasing, growing by an estimated 52% from 1990 to 2017 (Institute for Health Metrics and Evaluation 2018). The multifaceted sequelae of living with disability are broad ranging and impact affected persons’ psychosocial, socio-economic and physical well-being. This has been born out in literature, as studies have shown that persons with disabilities have decreased access to healthcare services, education and both entering and participating in the labour market, effectively creating a cyclical relationship between disability and poverty (Banks, Kuper & Polack 2017; Ganle et al. 2020; Moscoso-Porras, Fuhs & Carbone 2019; Payne, Mkandawire & Kohler 2013; Soltani et al. 2019; Trani et al. 2018; Yeo & Moore 2003). Many of these downstream effects of living with disability relate to a lack of accessibility – to physical places, opportunities and, more broadly, to inclusion, respect and dignity.

The effects of disability are magnified in resource-poor nations, such as Malawi, a country in southeast Africa with the 12th lowest gross domestic product per capita in the world (The World Bank 2020) and a majority of its population living in rural areas (Central Intelligence Agency 2021). In 2018, 10.4% of the population was affected by disability (National Statistical Office, Malawi 2019), of which 13.0% did not have access to a healthcare facility and 89.0% of persons in need of assistive devices did not have access to them (United Nations 2019). In the Northern Region of Malawi, the least developed and most rural of the country’s three regions, the situation is worse: 19.0% of those living in the region are living with disability and 3.2% are living with a mobility impairment (relative to 2.8% nationally) (National Statistical Office, Malawi 2019). Whilst the economic consequences of disability in Malawi and other low- and middle-income countries have been studied (Backup 2009), the psychosocial consequences of these care gaps remain poorly defined in low-income rural settings such as the Northern Region of Malawi.

In partnership with the nonprofit Crutches 4 Africa (n.d.), more than 1800 mobility-assistive devices were donated to the government-sponsored organisation Malawi Against Physical Disabilities in an effort to help alleviate the burden of mobility impairment throughout the Northern Region of Malawi. In conjunction with this donation effort, device recipients were surveyed in an attempt to better define the effects of their pre-existing physically limiting disabilities on their life satisfaction. Mobility clinics were organised to assess mobility impaired adults for device fitting and to measure their baseline functional mobility and life satisfaction. The aims of this study are to: (1) define the burden and presenting aetiologies of mobility impairment in the rural Northern Region of Malawi and (2) assess the relationship between physical disability, life satisfaction and access to mobility aids.

## Methods

### Design and study setting

Over a 10-day period in July 2019, observational, cross-sectional surveys of 251 adults with mobility impairments were conducted at mobility clinics in the Rumphi, Mzimba and Nkhata Bay Districts of the Northern Region of Malawi. Ethical approval for this research was obtained both by the University of California Los Angeles and the Malawian National Health Science Research Committee.

The study team was composed of a research team from the United States of America (USA) and physiotherapists and volunteers from the government-sponsored organisation Malawi Against Physical Disabilities. The study population was selected using convenience sampling, in which persons with disabilities who were able to attend the mobility clinics were asked to participate in the study. Employees of Malawi Against Physical Disability and local volunteers advertised the dates and times of the mobility clinics at which donation distribution and research would be conducted. Adults with mobility impairments that attended mobility clinics were examined and assigned an appropriate mobility device by a licensed physiotherapist. Results of pre- and post-assessment device usage were recorded.

Inclusion criteria were that research participants had to be adults (18 + years old) with mobility impairments – defined as any limitation in walking. Clinic attendees that met these criteria were invited to complete a demographic questionnaire and a series of globally validated surveys to qualify their degree of mobility impairment, physical activity and life satisfaction. Survey respondents were informed beforehand that their participation in the study was optional and would have no bearing on their receipt of an assistive device. Verbal consent was obtained by the research team, and surveys were conducted verbally with the help of interpreters. Attendees were excluded if they were over 18 years of age, had intellectual impairments precluding survey comprehension and/or completion and/or were unable to speak English, Chichewa, Chitumbuka or Tonga.

In total, 16 mobility clinics were organised in locations determined to minimise attendee travel and maximise accessibility to the dispersed populations of the three included districts. During the study period, 257 adults were assessed for device distribution, 251 of whom agreed to participate in the study. Research participants who completed the demographic survey only were excluded from the analysis of mobility impairment and life satisfaction. The longitudinal effect of distributing mobility devices was not studied, as the coronavirus disease 2019 (COVID-19) pandemic limited follow-up.

### Measures

A demographic survey designed by the study team was used to collect data on each respondents’ age, gender, highest level of education, employment status, and disability history. This survey also captured the mode of transportation and time needed to travel to their respective mobility clinics.

The Global Physical Activity Questionnaire (GPAQ) and the Washington Group Extended Set Questions on Disability were utilised to quantify physical disability and mobility impairment among participants. The GPAQ is a World Health Organization-developed tool that catalogues respondents’ weekly activity levels in the contexts of work, daily travel and/or recreation. Time spent actively is weighted based on the reported vigorousness of the activity and summed to yield a score representative of each respondent’s physical activity levels. The summary score is provided using metabolic equivalent minutes per week, represented in this study as metabolic equivalent minutes per day (World Health Organization 2002). The Washington Group Extended Set Questions on Disability is a survey developed by the Washington Group, the Budapest Initiative and the Economic and Social Commission for Asia and the Pacific as a standardised and validated tool to assess disability across culturally and socio-economically diverse populations. The survey is broken down into various functional domains. The mobility domain was utilised in this study because it was used in the Malawi Population and Housing Census and allows for comparability between this study’s data and that collected from the census (National Statistical Office, Malawi 2019; Washington Group on Disability Statistics 2019).

Two patient-reported outcomes measurement information systems surveys, developed by the National Institute of Health and independently validated, were utilised to measure life satisfaction among participants: (1) satisfaction with participation in social roles – Short Form 8a (PROMIS Health Organization 2016) and (2) general life satisfaction – Short Form 5a (Hahn et al. 2016; PROMIS Health Organization 2017; Vaughan, Mulcahy & Fitzgerald 2020). The satisfaction with participation in social roles survey was used to measure participants’ satisfaction with their ability to perform daily tasks and fulfil familial roles. The general life satisfaction – Short Form 5a survey was used to measure participants’ overall life satisfaction. For both quality-of-life measures, raw scores were standardised using patient-reported outcomes measurement information systems survey-specific scoring scales. Higher summary scores indicate greater participant satisfaction.

### Data analysis

Responses were summarised using mean and standard deviation, frequency and/or relative frequency as appropriate. Correlations between mobility measures and quality of life measures were assessed using Spearman’s correlation coefficient, and 95% confidence intervals (CI) were used. Statistical analyses were computed using STATA/IC 16.1, and *p*-values < 0.05 were considered statistically significant.

### Ethical considerations

The University of California, Los Angeles Institutional Review Board (UCLA IRB) has approved the above referenced study. UCLA’s Federalwide Assurance (FWA) with the Department of Health and Human Services is FWA00004642.

## Results

### Demographics

Of the 251 enrolled participants, 193 completed all four surveys. The remaining 58 completed only the demographic survey. The mean age of respondents was 64.7 years; 49.0% were female (Table 1). Meanwhile, 70.5% of respondents reported primary school as their highest level of schooling, whilst 11.2% reported not attending any school at all.

**TABLE 1:** Summary of demographics, state of disability, and life satisfaction in the sample population.

Variables	Rumphi District	Mzimba District	Nkhata Bay District	Total
Participants	138	43	70	251
Clinics	6	5	5	16
Average age	73.0	51.9	56.3	64.7
Disability length (total in years)	9.6	19.3	23.9	15.3
Disability length (as a % of life)	14.8	44.0	50.1	29.6
Years with an assistive device	6.9	15.3	16.3	10.9
Sedentary min per day	349.0	350.0	421.0	369.6
Metabolic equivalent min per day	585.9	1468.2	689.5	806.2
Satisfaction with participation in social roles	42.0	43.6	39.8	41.7
General life satisfaction	42.3	40.6	38.2	40.8

### Disability burden

The mean duration of disability at the time of surveillance was 15.2 years. Adjusting for age, the mean percentage of life spent with a mobility impairment was 29.6%. Of the Washington Group Extended Set Questions on Disability respondents, 23.4% reported having some difficulty walking, whilst 61.4% reported having a lot of difficulty and 11.2% reported being unable to walk (Table 2). Specific GPAQ results, including metabolic equivalent minutes per day and sedentary minutes per day, are summarised in Table 1.

**TABLE 2:** Difficulty walking, as measured by the *Washington Group Extended Set Questions on Disability*, in the sample population compared to the general population, by district.

Difficulty walking	Rumphi			Mzimba			Nkhata Bay			Total/Northern Region		
	Count	%	Census %†	Count	%	Census %†	Count	%	Census %†	Count	%	Census %†
No difficulty	7	7.1	-	1	2.4	-	0	0.0	-	8	4.1	-
Some difficulty	24	24.2	83.6	10	23.8	84.0	12	21.4	81.9	46	23.4	83.6
A lot of difficulty	58	58.6	13.5	27	64.3	12.8	36	64.3	15.5	121	61.4	13.5
Cannot do	10	10.1	2.9	4	9.5	3.2	22	11.2	2.6	22	11.2	2.9

† , As reported by the Malawi Population and Housing Census (National Statistical Office, Malawi 2019).

The burden of specific physical disabilities captured is summarised in Table 3. Of the 251 participants, 215 reported contributing conditions. Many of these respondents reported more than one contributory condition. Of these 215 participants, 196 (91.2%) were affected by joint-related and/or degenerative conditions. The average age of the participants in this group was 72.4 years old, whilst those whose disabilities were of a traumatic or neurological and/or congenital aetiology had an average age of 55.7 and 52.7 years, respectively. Among all participants, the most common contributor was knee pain (*n* = 64; 26.8%), the most common neurologic aetiology was poliomyelitis (*n* = 22; 10.2%) and the most common traumatic aetiology was lower extremity amputation (*n* = 12; 5.6%) (Table 3).

**TABLE 3:** Burden of musculoskeletal disease.

Etiology†	Count	Prevalence (%) (*n* = 215)	Average age (years)
**Degenerative and/or joint-related**	205	95.3	71.3
Leg pain, unspecified	27	12.6	69.1
Knee pain	64	29.8	75.5
Hip pain	22	10.2	74.6
Foot or ankle pain	12	5.6	75.6
Lower back pain	9	4.2	77.9
Joint stiffness	11	5.1	74.0
Generalised muscle weakness	27	12.6	66.2
Instability	17	7.9	73.7
Abnormal gait	3	1.4	73.3
Leg length discrepancy	4	1.9	43.8
**Traumatic**	20	9.3	55.7
Lower extremity fracture	8	3.7	61.8
Traumatic lower extremity amputation	12	5.6	51.7
**Neurological and/or congenital**	57	26.5	53.6
History of poliomyelitis	22	10.2	52.4
History of cerebral palsy	11	5.1	32.4
History of cerebrovascular event	18	8.4	66.5
History of meningitis	2	0.9	47.5
Congenital malformation	9	4.2	45.6
History of spinal tuberculosis	4	1.9	64.0

† , Some respondents reported living with disability or disabilities secondary to multiple aetiologies.

### Mobility device history

Seventy-three point two percent (183/251) of respondents used or had used some sort of mobility device in the past, the most common of which included: (1) sticks (*n* = 64), (2) canes (*n* = 37), (3) axillary crutch(es) (*n* = 21) and (4) Lofstrand crutch(es) (*n* = 13). Post-assessment, more Lofstrand crutches were prescribed (*n* = 33) than axillary crutches (*n* = 28), increasing the relative rate of Lofstrand crutch use among crutch users from 38.2% to 54.1%. Similarly, 16 wheelchairs were prescribed post-assessment, whilst only nine respondents had wheelchairs pre-assessment. Pre- and post-assessment device distribution data can be found in Table 4.

**TABLE 4:** Pre- and post-assessment device usage.

Variable	None	Stick or cane	Lofstrand crutch(es)	Axillary crutch(es)	Wheelchair	Walker	Other
Pre-assessment device	66	101	13	21	9	0	2
Post-assessment device	4	82	33	28	16	26	3
% change	−93.90	−18.81	+153.8	+33.3	+77.8	-	-

Relative to the 103 GPAQ respondents who had not previously used a formal mobility device, the 70 respondents who had used formal devices reported 235.5% of the amount of metabolic equivalent minutes per day (95% CI: [139%, 333%], *p* = 0.006). Those who had used a formal mobility device also reported significantly higher satisfaction with participation in social roles (95% CI: [0.21, 6.67], *p* = 0.037) and equivocally higher general life satisfaction (95% CI: [−1.77, 3.84], *p* = 0.470).

### Mobility and life satisfaction

Higher physical activity scores (measured in metabolic equivalent minutes per day) were positively correlated with life satisfaction scores, as measured by both satisfaction with participation in social roles – Short Form 8a (Spearman’s rho 0.481; *p* < 0.0001) and general life satisfaction – Short Form 5a (Spearman’s rho 0.230; *p* < 0.001). Increased time spent sedentary was negatively correlated with both satisfaction with participation in social roles (Spearman’s rho −0.507; *p* < 0.0001) and general life satisfaction (Spearman’s rho −0.306; *p* < 0.0001) (Table 5). Age was negatively correlated with metabolic equivalent minutes per day (Spearman’s rho −0.345; *p* < 0.0001) and satisfaction with participation in social roles (Spearman’s rho −0.193; *p* < 0.01) but not with general life satisfaction.

**TABLE 5:** Correlations between physical activity and life satisfaction, further stratified by gender.

Variable	Satisfaction with participation in social roles		General life satisfaction	
	*n*	Spearman’s rho	*n*	Spearman’s rho
**Metabolic equivalent min/day**	189	0.481**	191	0.230*
Male	94	0.452**	97	0.144
Female	95	0.498**	94	0.305*
**Sedentary min per day**	189	−0.507**	192	−0.306**
Male	95	−0.189	95	−0.366*
Female	94	−0.619**	93	−0.400**

* , *p*-value < 0.001;

** , *p*-value < 0.0001.

On average, female GPAQ respondents had higher physical activity scores and sedentary activity scores than male participants, but neither were statistically significant. There was also no statistically significant difference in satisfaction with participation in social roles or general life satisfaction between genders.

## Discussion

The primary objectives of this study were to examine the current state of disability in the rural Northern Region of Malawi and to better define the relationship between physical activity and life satisfaction. In this sample population, a significant positive correlation was revealed not only between physical activity and life satisfaction but also between formal mobility device utilisation and physical activity, as well as formal mobility device utilisation and life satisfaction.

Literature focusing specifically on the association between mobility impairment and life satisfaction, especially in low-resource settings, is relatively limited, but this study’s results are generally concordant with previously published data that demonstrate the negative impact that mobility impairment has on various psychosocial outcomes. Researchers studying 4245 persons aged 45 years and older in China found a significant correlation between mobility impairment and decreased life satisfaction, which was attributed in part to decreased social engagement in the affected population (Li & Loo 2017). Studies conducted in high-income countries have shown similar results. Using a national longitudinal survey focused on the biopsychosocial aspects of health and well-being, researchers studying the population of the USA found that mobility impairment was not only associated with decreased life satisfaction but also with negative affect, pessimism and decreased coping skills (Na & Singh 2021).

Literature has also shown that the provision of assistive devices like prostheses and/or orthoses can have broad and multifactorial effects on improving the quality of life of persons with disability. Prostheses and orthoses were found to improve psychological, environmental and physical health quality of life in South India (Magnusson et al. 2019) and physical activity in South Africa (Pienaar & Visagie 2019). Similarly, assistive devices were shown to increase employment and school attendance in Southern Africa (Eide & Øderud 2009) and environmental health in Indonesia (Toro, Eke & Pearlman 2016). This is in accordance with the results presented in this article, suggesting that treating mobility impairment has the potential to also treat its downstream sequelae. However, it is important to note that this topic remains under-studied, particularly in resource-limited nations, as a scoping review article of 430 publications on the provision of prostheses or orthoses in low-resource environments included only six studies with metrics related to quality of life (Ikeda et al. 2014).

Whilst this study’s sample population is not representative of the diverse populations throughout sub-Saharan Africa, similar psychosocial sequelae of disability likely exist in other low-resource settings. Especially in the developing world, where the rural majority often relies on smallholder farming, mobility is vital to successful social and economic integration. Thus, better understanding the context-specific impacts of mobility impairment and deploying appropriate strategies to address underlying conditions with mobility-assistive devices has the potential to increase physical activity and social and economic engagement (Bertrand et al. 2017; Kabiri et al. 2018; Rosso et al. 2013). Whilst the international community has placed increased emphasis on the rights of disabled persons (United Nations 2007, 2015), empathy alone cannot substitute for surveillance and treatment of physical disability in low- and middle-income countries that have historically borne a disproportionate share of the burden of disability because of resource and geographic limitations (Murray & Lopez 1997).

Device donation efforts, such as the one described in this article, may also be parlayed into health surveillance efforts in hard-to-reach rural populations. As was the case in this study, coupling health surveillance and/or cross-sectional research with donation initiatives can help incentivise clinic attendance and facilitate convenience sampling. This coupled method benefits respondents both by: (1) providing direct aid and (2) cataloguing the disease burden and needs of affected populations – allowing for more targeted, data-informed interventions in the future. Through this strategy, aid and research can be conducted in a mutually beneficial, needs-driven manner not possible through isolated aid or isolated research alone. This coupled strategy can be similarly employed for treatable disability related to vision and hearing deficits.

Whilst coupling research with aid provision facilitates needs-based closed-loop communication in target populations (Barstow et al. 2014), there are ethical implications of this strategy that must also be considered. In general, donation initiatives inherently create a power imbalance between the provider and recipient of the aid (Zarka, Farhat & Gidron 2018). Those completed in the absence of input from local stakeholders run the risk of neglecting community needs and cultural landscapes and can be wasteful and deleterious (Bauer 2017; McDonald et al. 2019). Recognising the power structures in place whilst conducting this study, the research team took steps to preserve autonomy and promote beneficence in the target population. To do this, local stakeholders – employees and volunteers from the organisation Malawi Against Physical Disabilities in conjunction with tribal leadership – were centrally involved with research planning and execution. Malawi Against Physical Disabilities oversaw the entirety of the donation process to ensure that the initiative safeguarded individual and community needs and beliefs. Using this locally based structure, efforts were made to avoid a common pitfall of donation initiatives – providing short-term benefit, not based on local context or needs, at the expense of a more long-term, sustainable solution (Newall et al. 2019).

Whilst this study adds to the growing body of literature related to physical disability and its psychosocial manifestations in low-resource environments, there are many relevant limitations to this study that should be considered. First, because of the impact of the COVID-19 pandemic, the research team was unable to reassess participants after receipt of mobility devices to measure the impact of device provision on participant mobility and life satisfaction. In addition, this study utilised convenience sampling, in which only persons with disabilities that were able to attend the mobility clinics were included in the study. Whilst efforts were made to select clinic sites that were centrally located and accessible to target populations, this sampling strategy may have impacted the results by precluding the participation of those unable to travel because of a higher degree of disability, geographic constraints and/or financial limitations. Additionally, the authors recognise that despite the efforts mentioned previously, power imbalances associated with the provision of aid may have led to some response bias among survey respondents. Lastly, it is also important to note that the study population is not nationally representative. In this study, 23.4% of Washington Group Extended Set Questions on Disability respondents reported having some difficulty walking, whilst 61.4% reported having a lot of difficulty and 11.2% reported being unable to walk. In comparison, the Malawi Population and Housing Census data reported that 83.6% of respondents have some difficulty walking, 13.5% have a lot of difficulty walking and 2.92% are unable to walk (National Statistical Office, Malawi 2019). This skew towards severe disability was expected given the use of convenience sampling but limits the study’s generalisability.

Whilst this study focused on physical disability as it relates to life satisfaction and access to mobility assistive devices, the biopsychosocial consequences of physical disability are far-reaching and extend into the domains of financial and psychological well-being. Future research with longitudinal follow-up is needed to better understand both the impact that mobility impairment has on the lives of persons with disabilities and the efficacy and ethics of coupling research and donation efforts.

## Conclusion

In summary, given the strong positive correlations found between: (1) physical activity and life satisfaction, (2) formal mobility device usage and physical activity and (3) formal mobility device usage and life satisfaction, increasing access to mobility assistive devices in undertreated populations is imperative. Such interventions have the power to increase mobility and subsequently improve the quality of life of persons with disabilities, likely by enabling home and workplace productivity and increasing access to social services like banking, education and healthcare. Moreover, dissemination of mobility devices and other adaptive equipment may be coupled with health-surveillance research, allowing for more data-driven targeted interventions in the future. As exemplified in this study, disability and mental health do not exist in isolation from one another. The impact of mobility impairment is multi-dimensional, and further research and evidence-based interventions should be prioritised to address the far-reaching physical and mental health inequities that stem from it.
